# Brief Education on Microvasculature and Pit Pattern for Trainees Significantly Improves Estimation of the Invasion Depth of Colorectal Tumors

**DOI:** 10.1155/2014/245396

**Published:** 2014-05-25

**Authors:** Joon Sung Kim, Bo-In Lee, Hwang Choi, Bong Koo Kang, Jong In Kim, Hae Mi Lee, Eun-Joo Im, Byung-Wook Kim, Sang-Woo Kim, Myung-Gyu Choi, Kyu Yong Choi

**Affiliations:** ^1^Division of Gastroenterology, Department of Internal Medicine, Incheon St. Mary's Hospital, The Catholic University of Korea, 665 Bupyeong-dong, Bupyeong-gu, Incheon 403-720, Republic of Korea; ^2^Division of Gastroenterology, Department of Internal Medicine, Seoul St. Mary's Hospital, The Catholic University of Korea, 222 Banpo-daero, Seocho-gu, Seoul, Republic of Korea

## Abstract

*Objectives*. This study was performed to evaluate the effectiveness of education for trainees on the gross findings identified by conventional white-light endoscopy (CWE), the microvascular patterns identified by magnifying narrow-band imaging endoscopy (MNE), and the pit patterns identified by magnifying chromoendoscopy (MCE) in estimation of the invasion depth of colorectal tumors. *Methods*. A total of 420 endoscopic images of 35 colorectal tumors were used. Five trainees estimated the invasion depth of the tumors by reviewing the CWE images before education. Afterwards, the trainees estimated the invasion depth of the same tumors after brief education on CWE, MNE and MCE images, respectively. *Results*. The initial diagnostic accuracy for deep submucosal invasion before education and after education on CWE, MNE, and MCE findings was 54.3%, 55.4%, 67.4%, and 76.6%, respectively. The diagnostic accuracy increased significantly after MNE education (*P* = 0.028). The specificity for deep submucosal invasion before education and after education on CWE, MNE, and MCE findings was 47.9%, 45.7%, 65.0%, and 80.7%, respectively. The specificity increased significantly after MNE (*P* = 0.002) and MCE (*P* = 0.005) education. *Conclusion*. Brief education on microvascular pattern identification by MNE and pit pattern identification by MCE significantly improves trainees' estimations of the invasion depth of colorectal tumors.

## 1. Introduction

Early colorectal cancers with submucosal invasion of 1000 *μ*m or less have very little risk of lymph node metastasis [1–3]. Tumors without deep submucosal invasion can be treated by endoscopic resection, whereas tumors with deep submucosal invasion should be treated with surgery. Thus, the estimation of depth is one of the most important steps for establishing therapeutic plans when presented with an early colorectal cancer-like lesion.

The depth of invasion can be estimated using the gross findings identified by conventional white-light endoscopy (CWE), the microvascular patterns identified by magnifying narrow-band imaging endoscopy (MNE), and the pit patterns identified by magnifying chromoendoscopy (MCE). The usefulness of MNE and MCE in distinguishing neoplastic from nonneoplastic colonic polyps [4–6] and in estimating the depth of invasion was already demonstrated by previous studies [7, 8], most of which involved a highly experienced endoscopist. Few studies have investigated the accuracy and the educational effect of these modalities in less experienced endoscopists estimating the depth of invasion.

Therefore, we performed this study to evaluate the effect of brief education on gross findings, microvascular patterns, and pit patterns for trainees estimating the invasion depth of colorectal tumors and to assess their interobserver agreements.

## 2. Methods

### 2.1. Preparation of the Electronic Images of Colorectal Tumors

All of the electronic photographs were recorded with magnifying endoscopes (CF-H260AZL; Olympus Co., Tokyo, Japan) by a highly experienced endoscopist (Bo-In Lee) at Incheon St. Mary's Hospital between January and December 2011. In total, 420 electronic photos (average of four images, each for CWE, MNE, and MCE) of 35 colorectal tumors were included to evaluate the effect of brief education on gross findings, microvasculature, and pit patterns for trainees estimating the invasion depth of colorectal tumors. All of the tumors had more than one gross characteristic suggesting the possibility of early colorectal cancers (Tis or T1 cancer): >2 cm, hardness, depression, surface nodularity, full expansion, a deformed adjacent wall, mucosal friability, the convergence of mucosal folds, erosion, and ulceration [9, 10]. Grossly obvious advanced cancers that had obstruction, infiltration, or large ulceration were excluded.

**Figure 1:**
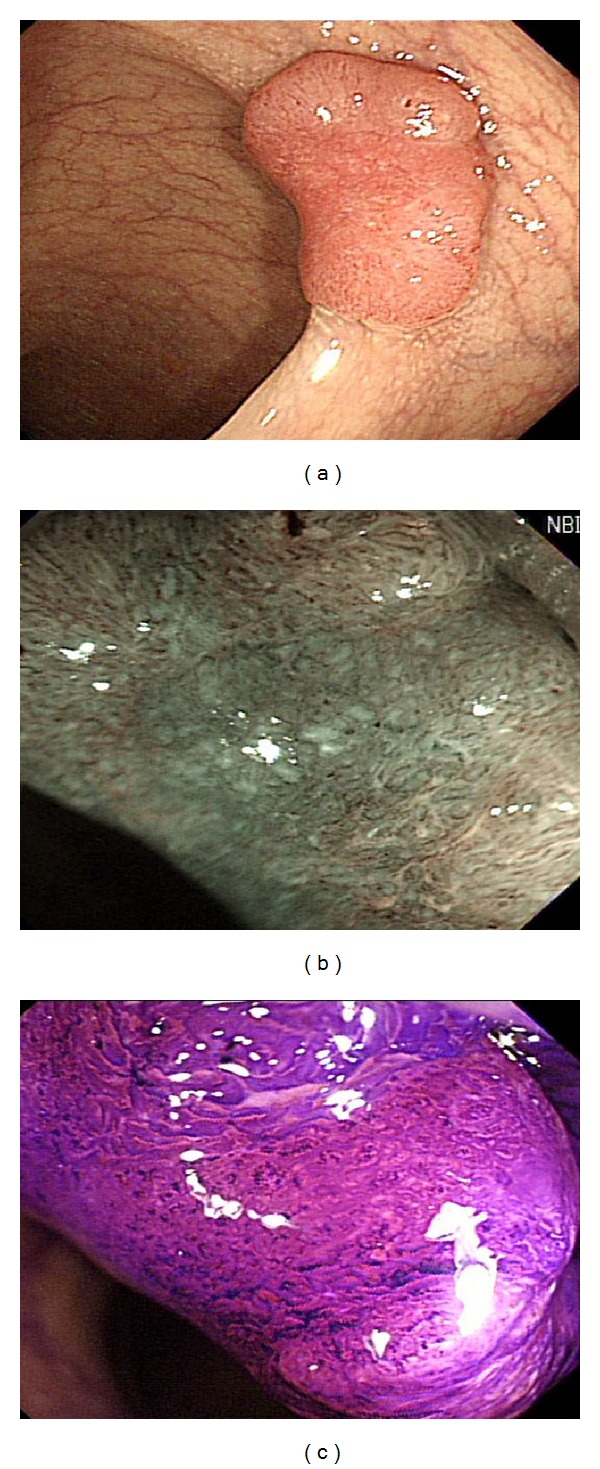
Electronic images of early colorectal cancer. (a) Conventional white-light colonoscopy image. (b) Magnifying narrow-band image. (c) Magnifying chromoendoscopy image.

When such a lesion was detected, the mucus and liquid feces on the surface of the lesion were washed away with simethicone-containing water. Endoscopic pictures were taken from as many angles as possible. The CWE image was recorded first, and the MNE and MCE images followed (Figure 1). Magnifying endoscopy was performed more carefully for depressed or erosive areas.

Chromoendoscopy was performed by spraying 0.4% indigo carmine. The application of 0.05% crystal violet was performed when the pit pattern was not obvious by indigo carmine staining. When the crystal violet was sprayed, an observation was performed after the dye had sufficiently stained the mucosa of the tumor. The location, size, and macroscopic type of each lesion were recorded. The lesions were classified macroscopically based on the criteria of the Paris classification [11]. Complete resection by endoscopic mucosal resection, endoscopic submucosal dissection, or surgery was attempted for all the tumors. All of the final histopathologic results were classified according to the Revised Vienna Classification of Gastrointestinal Neoplasia [12].

### 2.2. Estimation of the Invasion Depth by Trainees

Five trainees, who had performed colonoscopies for less than one year and had little experience in using MNE and MCE, were asked to review the electronic photographs of the tumors. The trainees were first presented with CWE images of the lesions and asked to estimate the presence of deep (≥1000 *μ*m) submucosal invasion. The trainees then received 40-minute education on gross findings suggesting deep submucosal invasion, such as hardness, depression, surface nodularity, full expansion, a deformed adjacent wall, mucosal friability, the convergence of mucosal folds, erosion, and ulceration, using electronic photographs and movie clips. Afterward, the same CWE images as in the initial test were presented to the trainees, who were asked to reevaluate the presence of deep submucosal invasion.

The trainees were then educated for 40 minutes on MNE findings suggesting deep submucosal invasion. The prediction of deep submucosal invasion by MNE was based on the Showa classification [13], which categorizes surface microvasculature as “normal,” “faint,” “network,” “dense,” “irregular,” or “sparse.” Lesions featuring a sparse microvascular pattern were assumed to have deep submucosal invasion. After education, the trainees were presented with MNE images of the same tumors and again asked to determine the presence of deep submucosal invasion.

Afterward, 40-minute education on MCE findings suggesting deep submucosal invasion was performed. Kudo's classification was used, and lesions with a V_N_ pit pattern were regarded as having deep submucosal invasion [7]. MCE images of the tumors were presented to the trainees, and estimation of the invasion depth was requested once again. The images of CWE, MNE, and MCE were all presented in the same order to the trainees and the trainees were not informed about their performance after each step.

Each session of education included typical CWE, MNE, or MCE images of lesions with or without deep submucosal invasion and other 33 exercise cases separate from the 35 test cases.

During the tests, only the age and sex of the patient were disclosed to the evaluators, and no discussion was permitted. The study protocol was approved by the institutional review board (OC12RISI0128).

### 2.3. Statistical Analysis

Diagnostic validities, including sensitivity, specificity, diagnostic accuracy, positive predictive value (PPV), and negative predictive value (NPV), were calculated by comparing the endoscopic estimation with the final histopathology. Significant differences were estimated using *χ* ^2^ tests. Interobserver agreement for each modality was determined using the intraclass correlation coefficient (ICC). An ICC value of ≤0.4 was regarded as poor agreement, 0.41–0.60 as fair agreement, 0.61–0.80 as good agreement, and >0.80 as excellent agreement. Statistical Analysis Software (SAS; Version. 9.1; SAS Institute, Cary, NC, USA) was used for all statistical analyses.

## 3. Results

### 3.1. Clinicopathologic Features of Colorectal Lesions

A total of 420 endoscopic images of 35 lesions (mean of four images per modality) from 35 patients were evaluated (Table 1). The mean patient age was 61 ± 10.5 years, and the male/female ratio was 2 : 3. The mean lesion size was 3.0 ± 1.0 cm. Of the 35 lesions, 22 were classified as laterally spreading tumors (LSTs), 10 as protruded types (Ip, Is), and three as flat types (IIa, IIb, and IIc). As for location, 14 (40%) were found in the right colon, nine (26%) in the left colon, and 12 (34%) in the rectum. Histopathological assessments included 13 low-grade neoplasias (low-grade adenoma/dysplasia), eight noninvasive high-grade neoplasias (high-grade adenoma/dysplasia, noninvasive carcinoma), and 14 invasive carcinomas (seven with submucosal invasion of 1000 *μ*m or less and seven with submucosal invasion more than 1000 *μ*m).

### 3.2. Diagnostic Validity for Deep Submucosal Invasion

Diagnostic validities, including specificity, diagnostic accuracy, and PPV, showed a tendency to be increased by education on CWE, MNE, and MCE findings suggesting deep submucosal invasion (Table 2). In particular, the diagnostic accuracy increased significantly after MNE education (55.4% versus 67.4%, *P* = 0.028). The diagnostic accuracy also increased after MCE education (67.4% versus 76.6%), although the difference was not statistically significant (*P* = 0.072). The specificity increased significantly after MNE (45.7% versus 65.0%, *P* = 0.002) and MCE (65.0% versus 80.7%, *P* = 0.005) education.

### 3.3. Assessment of Interobserver Agreement after Each Education

The ICC for the estimation of the depth of invasion by trainees before CWE education was 0.455. The ICC after CWE, MNE, and MCE education was 0.583, 0.535, and 0.554, respectively.

## 4. Discussion

It is challenging for less experienced endoscopists to estimate the invasion depth of colorectal tumors. Even less experienced endoscopists must decide on-site whether a tumor can be treated endoscopically or should be surgically resected, as a delayed scheduled polypectomy requires repeated bowel preparation and medical burdens. The inaccurate estimation of invasion depth can cause overly aggressive treatment, such as the surgical resection of tumors that can be treated endoscopically or unnecessary endoscopic treatment for tumors requiring surgical resection.

There are several methods for estimating the invasion depth of early colorectal cancers. The role of forceps biopsy is limited because the histopathology of biopsied specimens shows considerable discrepancies with the resected specimen in 40% of cases [14]. Several studies have reported the usefulness of endoscopic ultrasonography (EUS) for estimating the invasion depth [15–18]. However, the echoendoscope can be applied only to distal colorectal lesions, and the use of a miniprobe may be limited due to the probe's insufficient penetration depth for the estimation of large protruded tumors [19].

In previous studies, MCE and MNE have shown efficacy in the estimation of invasion depth of colorectal tumors. The type V_N_ pit pattern identified by chromoendoscopy has been shown to be an indicator of submucosal invasion in several studies [20–22], and the observation of microvascular patterns by NBI has also been shown to be effective for determining the presence of submucosal invasion [13, 23–25].

Experience is required for endoscopists to make a proper diagnosis using MCE and MNE. There is a learning curve for the proper analysis of microvasculature and pit patterns, and training is important for less experienced endoscopists to increase their diagnostic ability to differentiate between neoplastic and nonneoplastic polyps [26, 27]. However, no past studies have focused on estimation of the invasion depth of colorectal tumors by less experienced endoscopists.

In our study, the trainees could correctly diagnose the presence of submucosal invasion in only 54.3% of cases, and the accuracy after education on CWE findings was 55.4%, which was not a significant change. These diagnostic accuracies are very disappointing because the trainees chose either the absence or the presence of deep submucosal invasion, and the accuracies are not much different from the probability of a correct diagnosis by chance.

However, the trainees' diagnostic accuracy for invasion depth was increased significantly, from 55.4% to 67.4%, by only 40-minute education on MNE findings. The diagnostic accuracy also appeared to be increased to 76.6% by 40-minute education on MCE, although the difference did not reach statistical significance (*P* = 0.072). These results suggest that MCE and MNE are helpful for not only expert endoscopists but also less experienced endoscopists when estimating the invasion depth of colorectal tumors and that even brief education can improve diagnostic abilities.

The specificity was also increased significantly from 45.7% to 65.0% by MNE education and from 65.0% to 80.7% by MCE education. These results suggest that even less experienced endoscopists could better discriminate colorectal tumors without deep submucosal invasion from tumors with deep submucosal invasion if they were briefly educated on MNE and MCE findings. The discrimination of colorectal tumors without deep submucosal invasion is also important for the prevention of unnecessary surgery.

Interobserver agreement increased after education but did not increase beyond the fair agreement level for any of the modalities. It has been reported that MNE was comparable with MCE in estimating the invasion depth of early colorectal cancer, but there was greater interobserver variability using MNE [28]. Interestingly, interobserver agreement increased after education on CWE findings suggesting deep submucosal invasion; however, this agreement was not associated with an increase in diagnostic accuracy. Gross findings may have a limited role in the estimation of submucosal invasion, and estimation using only gross findings is even challenging for experts [29].

Various microvascular pattern classifications of colorectal lesions detected by MNE have been developed [13, 23, 30]. In this study, we used the Showa classification for microvascular patterns [13], and only a “sparse” pattern was used as an indicator of deep submucosal invasion. However, in the case of protruded and flat-elevated lesions, approximately one half of lesions with an “irregular” pattern were also shown to have deep submucosal invasion [31]. The reason why we regarded only a sparse pattern as an indicator of deep submucosal invasion is that we expected that simpler education would be more effective in improving the trainees' diagnostic accuracy. Additionally, we did not want to decrease specificity by also regarding an irregular pattern as an indicator of deep submucosal invasion. Recently, the NBI International Colorectal Endoscopic (NICE) classification has been proposed in an effort to develop a simple classification system to standardize the NBI observation criteria [32]. According to the NICE classification, the “irregular” pattern of the Showa can be classified as either “type II (low- or high-grade dysplasia, carcinoma in situ, and intramucosal carcinoma)” or “type III (deep submucosal invasive cancer).”

There are several advantages of using MNE in clinical practice. The conventional endoscopic view can be switched nearly instantaneously to the NBI view, and the use of NBI does not require any dye spraying. Standard observation by CWE followed by MNE for suspicious neoplastic lesions may be a good strategy for diagnostic colonoscopy [33]. Thus, we performed the tests in the order of CWE, MNE, and MCE, which is the practical order for observing colorectal lesions.

The first limitation of our study is the arbitrary selection of early colorectal cancer-like lesions by an expert endoscopist, which may have led to a selection bias. However, if the trainees were tested with consecutively detected colorectal polyps during screening colonoscopy, the diagnostic accuracy would have been much higher because tumors with deep submucosal invasion are not found so frequently during screening colonoscopy. The second limitation is that the trainees did not perform MNE or MCE by themselves, as an expert endoscopist recorded all of the images. However, we believe that the techniques of MNE and MCE are not difficult and that most trainees can learn the techniques in a short period of time under the guidance of an expert endoscopist. The third limitation of our study is that although the trainees' diagnostic accuracy and specificity increased after education, it did not lead to an increase in sensitivity. A decrease in sensitivity would inevitably lead to unnecessary trials for endoscopic resections of deep submucosal invasive cancers while a decrease in specificity would lead to unnecessary surgery for endoscopically resectable lesions. Therefore, we believe the increase in the trainees' diagnostic accuracy would still be valuable in clinical practice. Also, the number of cases included in our study and the number of trainees who participated in the education were small. Further studies involving more cases and more trainees are needed to confirm the results of our study.

In conclusion, trainees cannot accurately estimate the invasion depth of colorectal tumors using gross findings identified by CWE, and diagnostic accuracy is not improved by education on gross findings suggesting deep submucosal invasion. However, even brief education on microvascular patterns detected by MNE and pit patterns identified by MCE can significantly improve trainees' ability to diagnose the invasion depth of colorectal tumors. Advanced colonoscopy techniques, such as MNE and MCE, are helpful for not only expert endoscopists but also less experienced endoscopists to estimate the depth of invasion.

## 5. Conclusion

Brief education on microvascular pattern identification by magnifying narrow-band imaging endoscopy and pit pattern identification by magnifying chromoendoscopy can significantly improve trainees' ability to diagnose the invasion depth of colorectal tumors. Advanced colonoscopy techniques such as magnifying narrow-band imaging endoscopy and magnifying chromoendoscopy are helpful for less experienced endoscopists to estimate the invasion depth of colorectal tumors.

## Figures and Tables

**Table 1:** Patient characteristics and histopathological features of the tumors.

Patients	35
Male : female	14 : 21
Age, mean ± SD (years)	61.0 ± 10.5
Location	
Right colon	14
Left colon	9
Rectum	12
Size, mean ± SD (cm)	3.0 ± 1.0
Final pathology	
Low-grade neoplasia	13
Noninvasive high-grade neoplasia	8
Invasive carcinoma with submucosal invasion of 1000 *μ*m or less	7
Invasive carcinoma with submucosal invasion of more than 1000 *μ*m	7

SD: standard deviation.

**Table 2:** Estimation of deep submucosal invasion of colorectal tumors by trainees.

Education	Sensitivity (%, 95% CI)	Specificity (%, 95% CI)	Accuracy (%, 95% CI)	PPV (%, 95% CI)	NPV (%, 95% CI)
Before education	80.0 (63.1–91.6)	47.9 (39.4–56.5)	54.3 (46.6–61.8)	27.7 (19.3–37.5)	90.8 (81.5–96.1)
CWE	94.3 (80.8–99.3)	45.7 (37.3–54.3)	55.4 (47.7–62.9)	30.3 (21.8–39.8)	97.0 (89.5–99.6)
MNE	77.1 (59.9–89.6)	65.0 (56.5–72.9)*	67.4 (59.9–74.3)*	35.5 (24.9–47.3)	91.9 (84.7–96.5)
MCE	60.0 (42.1–76.1)	80.7 (73.2–86.9)*	76.6 (69.6–82.7)	43.8 (29.5–58.8)	89.0 (82.2–93.8)

PPV: positive predictive value; NPV: negative predictive value; CWE: conventional white-light endoscopy; MNE: magnifying narrow-band imaging endoscopy; MCE: magnifying chromoendoscopy.

*P < 0.05 when compared with the previous value.
